# Necessity of Hippocampal Neurogenesis for the Therapeutic Action of Antidepressants in Adult Nonhuman Primates

**DOI:** 10.1371/journal.pone.0017600

**Published:** 2011-04-15

**Authors:** Tarique D. Perera, Andrew J. Dwork, Kathryn A. Keegan, Lakshmi Thirumangalakudi, Cecilia M. Lipira, Niamh Joyce, Christopher Lange, J. Dee Higley, Gorazd Rosoklija, Rene Hen, Harold A. Sackeim, Jeremy D. Coplan

**Affiliations:** 1 Department of Psychiatry, College of Physicians and Surgeons, Columbia University Medical Center and New York State Psychiatric Institute, New York, New York, United States of America; 2 Department of Radiology, State University of New York at Brooklyn, Brooklyn, New York, United States of America; 3 Department of Psychiatry, State University of New York at Brooklyn, Brooklyn, New York, United States of America; 4 Brigham Young University, Provo, Utah, United States of America; 5 Department of Pathology and Cell Biology, College of Physicians and Surgeons, Columbia University Medical Center and New York State Psychiatric Institute, New York, New York, United States of America; 6 Macedonian Academy of Sciences and Arts, Skopje, Macedonia; RIKEN Brain Science Institution, Japan

## Abstract

**Background:**

Rodent studies show that neurogenesis is necessary for mediating the salutary effects of antidepressants. Nonhuman primate (NHP) studies may bridge important rodent findings to the clinical realm since NHP-depression shares significant homology with human depression and kinetics of primate neurogenesis differ from those in rodents. After demonstrating that antidepressants can stimulate neurogenesis in NHPs, our present study examines whether neurogenesis is required for antidepressant efficacy in NHPs.

**Materials/Methodology:**

Adult female bonnets were randomized to three social pens (N = 6 each). Pen-1 subjects were exposed to control-conditions for 15 weeks with half receiving the antidepressant fluoxetine and the rest receiving saline-placebo. Pen-2 subjects were exposed to 15 weeks of separation-stress with half receiving fluoxetine and half receiving placebo. Pen-3 subjects 2 weeks of irradiation (N = 4) or sham-irradiation (N = 2) and then exposed to 15 weeks of stress and fluoxetine. Dependent measures were weekly behavioral observations and postmortem neurogenesis levels.

**Results:**

Exposing NHPs to repeated separation stress resulted in depression-like behaviors (anhedonia and subordinance) accompanied by reduced hippocampal neurogenesis. Treatment with fluoxetine stimulated neurogenesis and prevented the emergence of depression-like behaviors. Ablation of neurogenesis with irradiation abolished the therapeutic effects of fluoxetine. Non-stressed controls had normative behaviors although the fluoxetine-treated controls had higher neurogenesis rates. Across all groups, depression-like behaviors were associated with decreased rates of neurogenesis but this inverse correlation was only significant for new neurons in the anterior dentate gyrus that were at the threshold of completing maturation.

**Conclusion:**

We provide evidence that induction of neurogenesis is integral to the therapeutic effects of fluoxetine in NHPs. Given the similarity between monkeys and humans, hippocampal neurogenesis likely plays a similar role in the treatment of clinical depression. Future studies will examine several outstanding questions such as whether neuro-suppression is sufficient for producing depression and whether therapeutic neuroplastic effects of fluoxetine are specific to antidepressants.

## Introduction

Major depression is consistently associated with decreased hippocampal volumes and deficits in hippocampus-dependent cognition [1], [2]. Some of these deficits may reflect structural changes in the hippocampal dentate gyrus. In preclinical studies, factors that predispose to depression, such as social stress [3], [4], maternal neglect [5], and drug abuse [6] decrease rates of new neuron formation (neurogenesis) in the dentate gyrus and cause cell atrophy and death in the CA1/CA3 region of the adult rodent hippocampus. Interventions that ameliorate major depression, including antidepressant medications, electroconvulsive therapy (ECT) [7], exercise, and environmental enrichment [8] stimulate dentate gyrus neurogenesis. These findings led to the hypotheses that suppression of neurogenesis leads to depression, and that stimulation of neurogenesis is required for treating depression [9], [10]. Despite generating widespread interest, this hypothesis is based mainly on indirect evidence derived mostly from rodents. A major limitation of rodent studies is that the phenomenological complexities of major depression are not evident in lower mammals. In addition, the speed and extent of neuronal maturation and the proliferation of neuronal precursor cells in the primate hippocampus is almost 10-fold less than in rodents [11]. Since there are no established methods of non-invasively detecting neurogenesis in humans, terminal studies of nonhuman primates are the best available options to examine the clinical relevance of these findings. Macaque monkeys are available for research in larger numbers than apes, and they display a richer repertoire of affective behaviors than New World monkeys. Bonnet macaques, in particular, form strong peer attachments that can be disrupted to produce plausible, core symptoms of depression [12], [13].

In the two previous studies related to neurogenesis in primates, hippocampal neurogenesis was suppressed in both adult marmoset monkeys exposed to acute intruder stress [14] and in juvenile rhesus macaques exposed to acute prenatal stress [15]. We reported that treatment with electroconvulsive stimulation (ECS), the pre-clinical equivalent of antidepressant electroconvulsive therapy (ECT), stimulated hippocampal cell proliferation and neurogenesis in adult bonnet macaques [16]. In the current study, we examined whether the therapeutic behavioral effects of antidepressant treatment required the induction of neurogenesis in adult bonnet macaques.

## Methods

### Ethics statement

All animal work has been conducted according to relevant national and international guidelines. In accordance with the recommendations of the Weatherall report, “The use of non-human primates in research.” the following statement to this effect has been included to document the details of animal welfare and steps taken to ameliorate suffering in all work involving non-human primates: This work was conducted at the Nonhuman Primate Facility of the State University of New York Downstate Medical Center with permission from its Institutional Animal Care and Use Committee (IACUC) The protocol number is 01-217-04, approved on 12/16/04. The welfare of the animals conformed to the requirements of *National Institutes of Mental Health (NIMH)*. All animals were housed in pens exceeding the stipulated sizes requirements. Animals were maintained in large group houses under 12-hour dark and light cycles, and were given access to food and water *ad libitum*. Animals were engaged with a variety of psychologically enriching tasks. No animal was physically harmed or knowingly exposed to potential infection.

Subjects and interventions: Adult female bonnet macaques were matched based on age, weight, social rank, and timing of menstruation, and randomized to a Control pen (n = 6) or a Stress pen (n = 6). Using the chronic stress paradigm developed in rhesus macaques [17], we exposed subjects in the Stress pen to social isolation for two days followed by social reunion on the remaining 5 days, repeated for a total duration of 15-weeks. The monkeys in the Control pen remained in social housing for those 15-weeks. During this period, half the subjects in each pen (Control-Drug and Stress-Drug groups) were treated with the selective serotonin reuptake inhibitor (SSRI), fluoxetine. In order to minimize the stress of administration, we used Prozac-weekly preparation (Eli Lilly Corp.), at a dose of 13.5 mg/kg infused via nasogastric tube (NGT) under sedation (ketamine 5 mg/kg and xylazine 1 mg/kg), once per week for 15-weeks. This dose was equivalent to a daily dose of 2 mg/kg of the drug. The remaining half (Control-Placebo and Stress-Placebo) received the same treatment with saline placebo via NGT. All groups were injected with the thymidine analog bromodeoxyuridine (BrdU) (100 mg/kg/day, I.V.) under ketamine/xylazine sedation for 5 days during week-7 of interventions (Stress/Control+Drug/Placebo) and then sacrificed by transcardiac perfusion with normal saline (500 ml/kg) followed by 4% paraformaldehyde (500 mg/kg) under deep anesthesia with pentobarbital (15 mg/kg, I.V.) on week-16 of interventions.

In a follow-up study, a social group of female bonnet macaques (Radiation-Stress-Drug group) were anesthetized with ketamine and xylazine, and given bilateral temporal lobe irradiation at a dose of 20Gy (n = 2) or 30Gy (n = 2) fractionated into 10 treatments over 2-weeks. This regimen was based on rodent studies, where irradiation effectively suppressed neurogenesis without causing significant necrosis or behavioral side effects [18], [19], [20]. A control group received ‘sham’ irradiation (anesthesia only) (n = 2). A Philips RT250 X-ray machine provided an X-irradiation beam (250 kVp, 15 mA, 0.4 cm Thoreau's filter, HVL = 0.3 mm^3^, 50 cm FSD (Focal Spot to Skin Distance). A customized shield made of cerabend (a lead alloy) was placed to cover the head with a large exterior 3.7 cm×1.8 cm rectangular hole (with rounded edges) that was collimated into a small internal field 2 cm in diameter. The X-ray beam was intended to target the entire hippocampus while sparing adjacent temporal lobe structures and the optic chiasm. Before starting irradiation, a plain film X-ray image was taken to confirm the positioning of the beam. After 2-weeks of irradiation or sham treatment, the subjects rested for 3-weeks to allow the acute effects of irradiation to dissipate as per previous rodent research [19]. Next, these monkeys were exposed to 15-weeks of repeated separation stress and concurrent antidepressant treatment with fluoxetine, injected with BrdU on week-7 and sacrificed on week-16, using the same methods described above.

Note: Since high- and low-dose irradiation had equivalent effects, the four irradiated animals were collapsed into one Radiated-Stress-Drug group (n = 4). Similarly, the sham-irradiated animals were collapsed into the Stress-Drug group (n = 5) because they were exposed to similar experimental conditions. Table 1 presents a schematic diagram of the study design.

**Table 1:** Study Design: Schematic illustrating study design and schedule.

Study Design					
Time	2 wks	3 wks	15 wks	Sacrifice	Histology
Group	Baseline		Control & Placebo (n = 3)		
			Control & Drug (n = 3)		
	Baseline		Stress & Placebo (n = 3)		
			Stress & Drug (n = 5)		
	XRT	Rest	Stress & Drug (n = 4)		

### Behavioral Ratings

**Figure 1:**
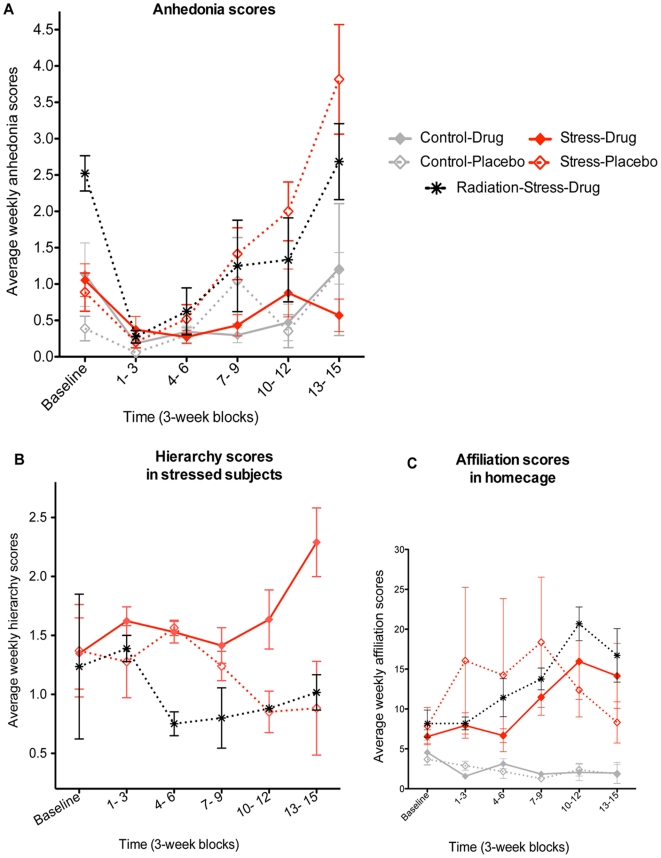
Behavioral Results. a. Anhedonia scores. Anhedonia scores showed an effect of group (F_4,65_ = 5.6, p = 0.0001), time (F_5,65_ = 17.1, p<0.0001), group and time interaction (F_20,65_ = 3.4, p = 0.0001). Bonferroni post-hoc tests showed that Stress-Placebo and Radiation-Stress-Drug groups had greater anhedonia compared to Control-Drug, Control-Placebo, and Stress-Drug groups by 13–15 weeks (p<0.01). Error bars represent standard error of mean. b. Hierarchy scores in stressed subjects. Hierarchy (defined as the difference between dominance and subordinance scores) showed an interaction between group and time (F_10, 35_ = 2.1, p = 0.04). Bonferroni post-hoc tests showed that the Stress-Drug group had higher hierarchy scores compared to Stress-Placebo (p<0.01) and Radiation-Stress-Drug (p<0.05) by week 13–15. The controls showed no changes in hierarchy over the 15 weeks (Fig.S1). c. Affiliation scores in homecage. Affiliation (physical contact and huddling) showed an effect of group (F_4, 65_ = 4.1, p = 0.023) and time (F_5, 65_ = 2.6, p = 0.033) and a group by time interaction (F_20, 65_ = 2.4, p = 0.005). Bonferroni post-hoc tests showed that the stressed subjects (Stress-Placebo, Stress-Drug, and Radiation-Stress-Drug) had greater affiliation compared to non-stressed subjects (Control-Drug and Control-Placebo) (p<0.05) during most of weeks 1–15.

Home-cage behaviors were quantified through one-way mirrors by trained raters (inter-rater correlation coefficient, ICC>0.96) for 3-days a week. The frequency of 40 behaviors typical to bonnet macaques [13] was scored for each animal at 30-second intervals per session. These behaviors were then collapsed into seven subscales determined *a priori* (Table 2). On the final week of observations (week-15), all animals were subjected to a single (60-seconds) exposure to a masked human intruder based on previously established methods [15]. Behavioral ratings were acquired during 5-minutes of intruder exposure and for 30-minutes post-exposure. The average behavioral scores for the seven behavioral subscales were analyzed in 3-week blocks in order to reduce fluctuations of individual sessions. Repeated measures analysis of covariance were conducted for each behavioral subscale, with baseline scores as covariates, and repeated measures involving time in 3-week time blocks over a total of 15-weeks. Effects within each behavioral domain were separately tested using Bonferroni post-hoc testing (p<0.05) (Fig. 1).

**Table 2:** Behavioral Categories: Set of 40 behaviors observed during home cage ratings were collapsed into eight subscales for behavioral analysis.

Domain	Behavior Symptoms
1. Affiliation	In close physical contact or proximity of other animals, huddling together.
2. Hedonia	Grooming others, toy play, sharing toys with others, playfully provoking dominants, embracing others, genital contact or sexual overtures.
3. Exploration	Explores cages walls or objects, watches doors for windows or doors for investigators.
4. Self- directed	Body scratching, shaking, grooming self.
5. Subordinance	Being chased, grabbed, pushed, bitten, or non-sexually mounted by dominant. Displaying subordinance postures or expressions (i.e., grimacing, yawning, lip smacking, presenting rear, raised tail, curled feet, rigid posture) when threatened or approached by dominant.
6. Dominance	Biting, chasing, growling, grabing, or non-sexual mounting another animal. Staring with canines, scowling, growling or taking threatening posture directed at subordinate. Inducing subordinance behavior in other animal's without over threat. Dominance displays (shaking cage).
7. Vigilance	Pacing or stereotypy (≥3 repetitive moves). Anxious posture (i.e., piloerection, freezing/rigid posture, raised tail, curled feet startle response, yawn) not induced by dominant animal.
8. Anhedonia	Alone and immobile, slumped or collapsed body posture, lack of purposeful eye movements or responsiveness to environmental stimuli, rejecting social advances.

### Postmortem studies

The left hippocampus was cut into 40 µm sections and immuno-stained to detect and quantify cell proliferation and neurogenesis rates using our previous methods [16]. Using standard peroxidase methods we determined the following: granule cells proliferating at the time of sacrifice (on week-15) identified by the expression of the mitotic marker Ki67 (mouse anti-Ki67 antibody, 1∶200; Vector Laboratories, Burlingame, CA); mitotic cells that took up BrdU at the time of injection (week-7) and survived until sacrifice (week-15) identified by BrdU labeling (mouse anti-BrdU, 1∶200; Becton Dickinson, San Jose, CA); and new neurons that were still immature, detected by the expression of the microtubule-associated protein doublecortin (DCX) [21], [22] (goat anti-doublecortin, 1∶200; Santa Cruz, CA). The secondary antibody was biotinylated anti-mouse IgG (1∶200; Vector Laboratories, Burlingame, CA), visualized with avidin-biotin complex solution (Vector Laboratories, Burlingame, CA), and diaminobenzidine (DAB; Sigma-Aldrich, St Louis, MO). Fluorescent labeling was used to detect the maturational fate of BrdU-labeled cells by co-labeling with markers of mature neurons, i.e., NeuN (mouse monoclonal anti-NeuN, 1∶200; Chemicon, Temecula CA), astroglia i.e., GFAP (mouse anti-GFAP, 1∶200; Dako, Carpinteria, CA) [16], and microglia i.e., Iba-1 (rabbit-anti-Iba-1, 1∶500; Dako, Carpinteria, CA) [23]. Additionally, we identified dentate gyrus neurons that were synaptically active by labeling for the immediate early gene c-Fos [24], [25] (rabbit anti-c-Fos, 1∶20,000; Calbiochem, Gibbstown, NJ) and new neurons that were at a hyperplastic stage of neuronal maturation by labeling for the NMDA receptor subunit II [26] (mouse anti-NMDA-subunit II, 1∶25; Millipore, Temecula, CA). The secondary antibodies were a mixture of Alexa 568-conjugated goat anti-rat IgG and Alexa 488-conjugated goat anti-mouse IgG (or anti-rabbit IgG for Iba-1) (1∶200; Invitrogen, Carlsbad CA). Since doublecortin (DCX) had not been previously used to detect neurogenesis in bonnet macaques, we confirmed its neuronal specificity in these animals by demonstrating co-labeling between DCX and the immature neuronal marker TUC-4 (rabbit anti-TUC-4, 1∶200; Millipore, Temecula, CA) and the absence of co-labeling with the astroglial marker GFAP [27]. Finally, we carefully inspected irradiated hippocampal sections for evidence of inflammation, apoptosis, or necrosis using hematoxylin and eosin (H & E) and Hoechst stains.

**Figure 2:**
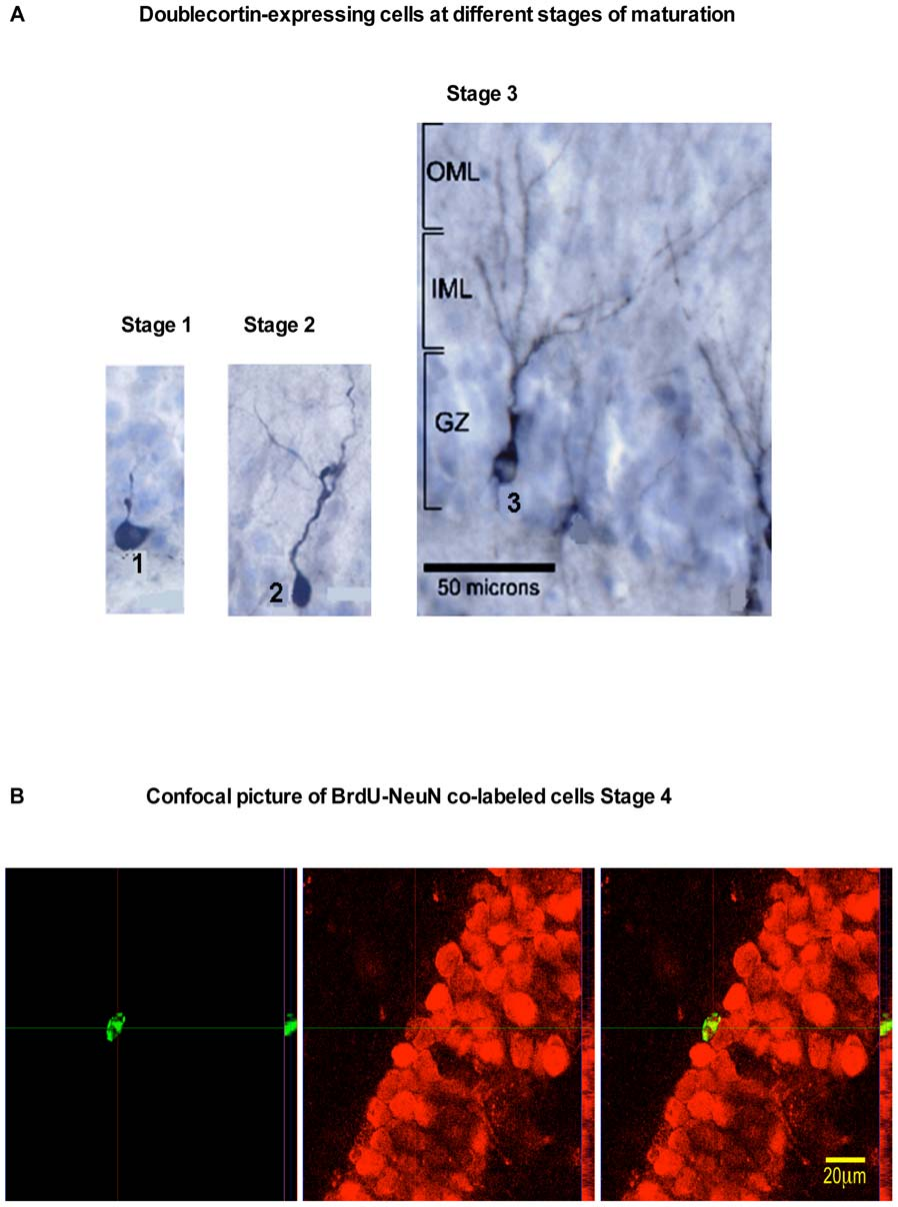
Images of Doublecortin-expressing new neurons at different stages of maturation and BrdU-NeuN co-labeled cells. a. Doublecortin-expressing cells in the SGZ at different stages of maturation. Stage 1. Immature neurons that lack dendrites, or have short dendrites that lack branches. Stage 2. Differentiating neurons with dendrites that have secondary branches and extend no further than the inner molecular layer. Stage 3. Neurons with dendrites that have tertiary branches and extend into the outer molecular layer. b. Confocal image of BrdU-NeuN co-labeled cells. Stage 4 BrdU-labeled cells (green) that also express the mature neuronal marker NeuN (red) with the overlayed images of NeuN and BrdU labeling (yellow).

The maturational stage of DCX-expressing cells was determined by comparing morphology with corresponding DCX-expressing neurons in the rodent hippocampus [28]. The post-mitotic age of these cells was estimated by comparing morphology to the appearance of BrdU-TUC-4 co-labeled hippocampal cells in adult rhesus macaques (phylogenetically close to bonnets) that were sacrificed at different time-intervals following BrdU injections [29]. Using this approach, DCX-expressing hippocampal neurons were divided into the following three different maturational stages. Stage 1: DCX-expressing neurons that lack dendrites or have rudimentary dendrites were estimated to be approximately 1–3 weeks-old because they corresponded to Stage 1 and early Stage 2 according to Ngwenya et al. [29] (Fig. 2A). Stage 2: DCX-expressing neurons with dendrites that had secondary branches extending no further than the inner molecular layer were estimated to be 3–5 weeks old because they corresponded to late Stage 2 according to Ngwenya et al. [29] (Fig. 2A). Stage 3: DCX-expressing neurons with mature dendrites that had tertiary branches and extended into the outer molecular layer were estimated at 6–7 weeks old because they corresponded to late Stage 3 according to Ngwenya et al. [29] (thus >5-weeks old) and because they did not co-label with BrdU (thus <8-weeks old) (Fig. 2A). Stage 4: BrdU-labeled cells that co-expressed NeuN were deemed to be 8–10 weeks old because BrdU was injected 8–9 weeks prior to euthanasia (Fig. 2B). DCX-expressing cells were further grouped based on anterior or posterior dentate gyrus location, given that there appears to be an anterior-posterior dichotomy in hippocampal function [30]. The anterior dentate gyrus was defined by coronal levels resembling Bregma-10.88 mm through Bregma-13.50 mm in the rhesus macaque brain atlas by Paxinos et al. [31]. The rest of the dentate gyrus was designated as the posterior segment.

All unambiguously labeled (e.g. BrdU, Ki67, DCX, Iba-1, c-Fos etc.) cells in every 20^th^ section (average of 15 per animal) of the left subgranular zone (SGZ) were conducted by two independent raters (ICC >0.90) who were blinded to animal identity. The SGZ was defined as a 50 µm band at the border of the granule cell layer (GCL) and hilus. The total number of counted cells was divided by the volume of the SGZ (length of SGZ in each section X 50 µm width X 40 µm thickness before post-mounting shrinkage of 50%–70%). Rates of labeled cells were expressed as a density per mm^3^ of the subgranular zone (SGZ). The volume of the granule cell layer (GCL) of left hippocampal sections stained with 4′, 6-diamidino-2-phenylindole (DAPI) (Sigma-Aldrich, St. Louis, MO) was estimated according to the Cavalieri Principle [32] using an Olympus BS 52 research microscope fitted with the Olympus DP72 camera. For all data, repeated measures ANOVA was conducted using irradiation, stress, and drug treatment as between-subject variables and counts of new neurons as the dependent measure with 2 within-subject factors: maturity, with 4 levels (Stages 1–4), and region, with 2 levels (anterior and posterior dentate gyrus regions).

## Results

### Behavioral Data

Exposure of monkeys that received placebo (Stress-Placebo group, n = 3) to repeated social separation stress led to gradual increases in behavioral scores for anhedonia (a behavioral composite of collapsed postures, inactivity, and blank stares) (Fig. 1A) and decreases in scores for hierarchy (total subordinate behaviors subtracted from total dominant behaviors) (Fig. 1B). Antidepressant treated cage-mates in the Stress pen (Stress-Drug, n = 3) did not show increases in anhedonia (Fig. 1A) or decreases in hierarchy (Fig. 1B). Affiliation scores (reflecting enhanced between-subject contact) were increased in stressed animals, irrespective of whether they were treated (Stress-Drug) or not (Stress-Placebo) compared to non-stressed controls (Control-Drug and Control-Placebo) (F_4,65_ = 4.1, p = 0.023) (Fig. 1C) throughout the 15-weeks of repeated separation-stress, with the greatest increases noted on days of reunion. Affiliation scores were also increased in the Stress groups compared to Controls in response to acute human intruder stress on week-15 (F_4,65_ = 4.1, p<0.05) (Fig. S1C). Non-stressed controls that received treatment (Control-Drug, n = 3) did not behaviorally differ from cage-mates that did not receive fluoxetine (Control-Placebo, n = 3) in terms of anhedonia (Fig. 1A) and affiliation scores (Fig. 1C).

The monkeys that underwent temporal lobe irradiation and were then exposed to separation stress and antidepressant treatment (Radiation-Stress-Drug group, n = 4) showed progressive increased anhedonia and decreased hierarchy scores compared to sham irradiated (anesthesia only) cage-mates (Stress-Drug, n = 2) (Fig. S1B). These behavioral changes in the irradiated animals closely paralleled changes in the Stress-Placebo group in terms of anhedonia (Fig. 1A) and hierarchy (Fig. 1B). By week 13–15 of stress, anhedonia scores showed an effect of group (F_4,65_ = 5.6, p = 0.0001) that resulted from increased scores in Stress-Placebo and Radiation-Stress-Drug subjects compared to Control-Drug, Control-Placebo, and Stress-Drug groups (p<0.01) per Bonferroni post-hoc tests (Fig. 1A). Hierarchy scores also showed an interaction between group and time (F_10,35_ = 2.1, p = 0.04) by week 13–15 with decreases in Stress-Placebo (p<0.01) and Radiation-Stress-Drug (p<0.05) groups compared to Control-Placebo subjects per Bonferroni post-hoc tests (Fig. 1B). The only exception was a transient increase in anhedonia scores in the Radiation-Stress-Drug group immediately after the completion of irradiation (XRT) in the absence of stress (Fig. 1A). The non-stressed Controls showed no increases in anhedonia (Fig. 1A) or changes in hierarchy over 15-weeks (Fig. 1B and Fig. S1A). There were no significant group differences in any of the other behavioral categories, i.e., self-stimulation, environmental exploration, and vigilance (data not shown), and there were no manifestations of physical distress or weight change.

### Histological results

**Figure 3:**
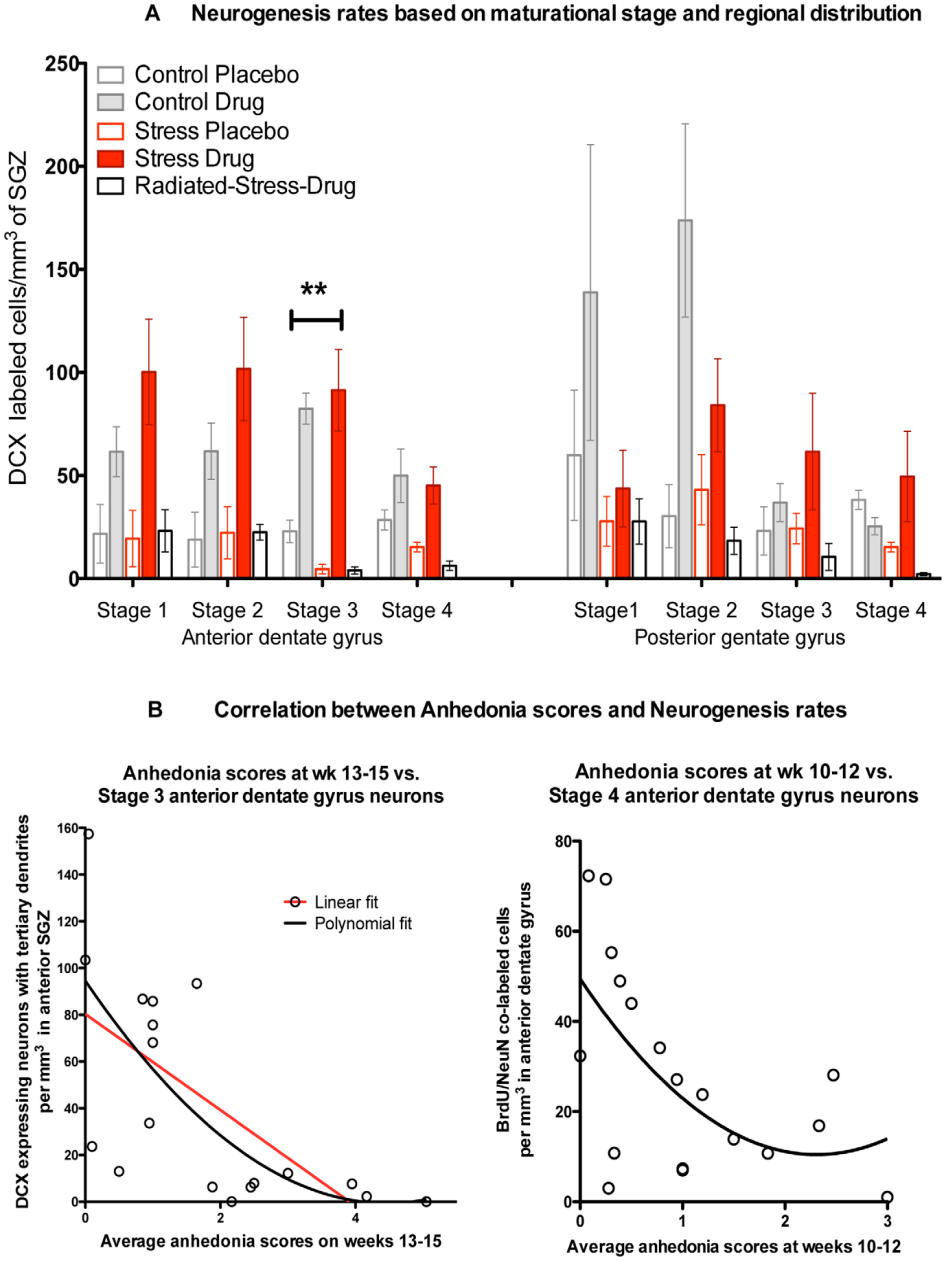
Neurogenesis rates. a. Neurogenesis rates based on maturational stage and regional distribution Neurogenesis rates at the three maturational stages were divided based on location in the anterior (left panel) or posterior (right panel) dentate gyrus. Multivariate analysis conducted on these six subdivisions showed an overall effect of experimental group (F_4,5_ = 90.01, p<0.0001). Univariate analysis of experimental group effects for each of the six sub-divisions of neurogenesis rates revealed significant effects only for Stage 3 neurons in the anterior dentate gyrus (F_4,5_ = 22.25, p<0.0001). Bonferroni post-hoc tests showed that only Stage 3 anterior dentate gyrus neurons were reduced in both depressive groups (Stress-Placebo and Stress-Drug-Radiation) compared to non-depressed groups (p<0.05). Data were Log_10_ transformed for statistical analysis to correct for uneven variance. b. Correlation between anhedonia scores and neurogenesis rates. Multiple regression analysis was conducted between anhedonia scores obtained at 5 different observation periods and the 6 subdivisions of neurogenesis rates. Anhedonia rates at the final observation period (weeks 13–15) inversely correlated *only* with Stage 3 anterior dentate gyrus neurons (r^2^ = 0.52, p<0.001). Since the regression curve appeared non-linear, a second degree polynomial fit was found to be the best (r^2^ = 0.67, p<0.0001). Anhedonia rates at weeks 10–12 inversely correlated with Stage 4 anterior dentate gyrus neurons (r^2^ = 0.28, p = 0.02). Likewise, a second-degree polynomial fit was found to be the best (r^2^ = 0.34, p<0.01).

Total neurogenesis rates, represented by the density of DCX-expressing cells in the subgranular zone, were increased in the Stress-Drug and Control-Drug groups and decreased in the Stress-Placebo and Radiation-Stress-Drug group, compared to rates in the Control-Placebo group (p<0.05) (Fig. 3A). Repeated measures ANOVA showed within-subjects interactions of neuronal maturity (stages 1–3) and region (anterior or posterior) that were of borderline significance (F_2,4_ = 3.3, p = 0.05). Between-subject effects were significant for drug (F_13,91_ = 26.6, p<0.001) and irradiation (F_13,91_ = 19.4, p = 0.001), but not for stress (F_13,91_ = 0.6, p = 0.45). When examining between-subject effects of maturity and region, there were significant interactions of region, maturity, and stress (p = 0.006) and region, maturity, and drug (p = 0.04). Bonferroni post-hoc tests showed that between-subjects effects were only significant for Stage 3 of maturity (in DCX-expressing cells) and the anterior region of the dentate gyrus (p<0.01) (horizontal bar in Fig. 3A). Compared to the Control-Placebo group, fluoxetine treatment increased Stage 3 anterior dentate gyrus neurons in the Stress-Drug and Control-Drug groups by 250% and 300%, respectively, while these rates were suppressed in the Stress-Placebo and Radiation-Stress-Drug groups by 84% and 90%, respectively (Fig. 3A). Further characterization showed that DCX-expressing granule cells at Stage 3 of maturity did not co-label with BrdU, NeuN, c-Fos, or the NR2B subunit of the NMDA receptor (not shown).

**Figure 4:**
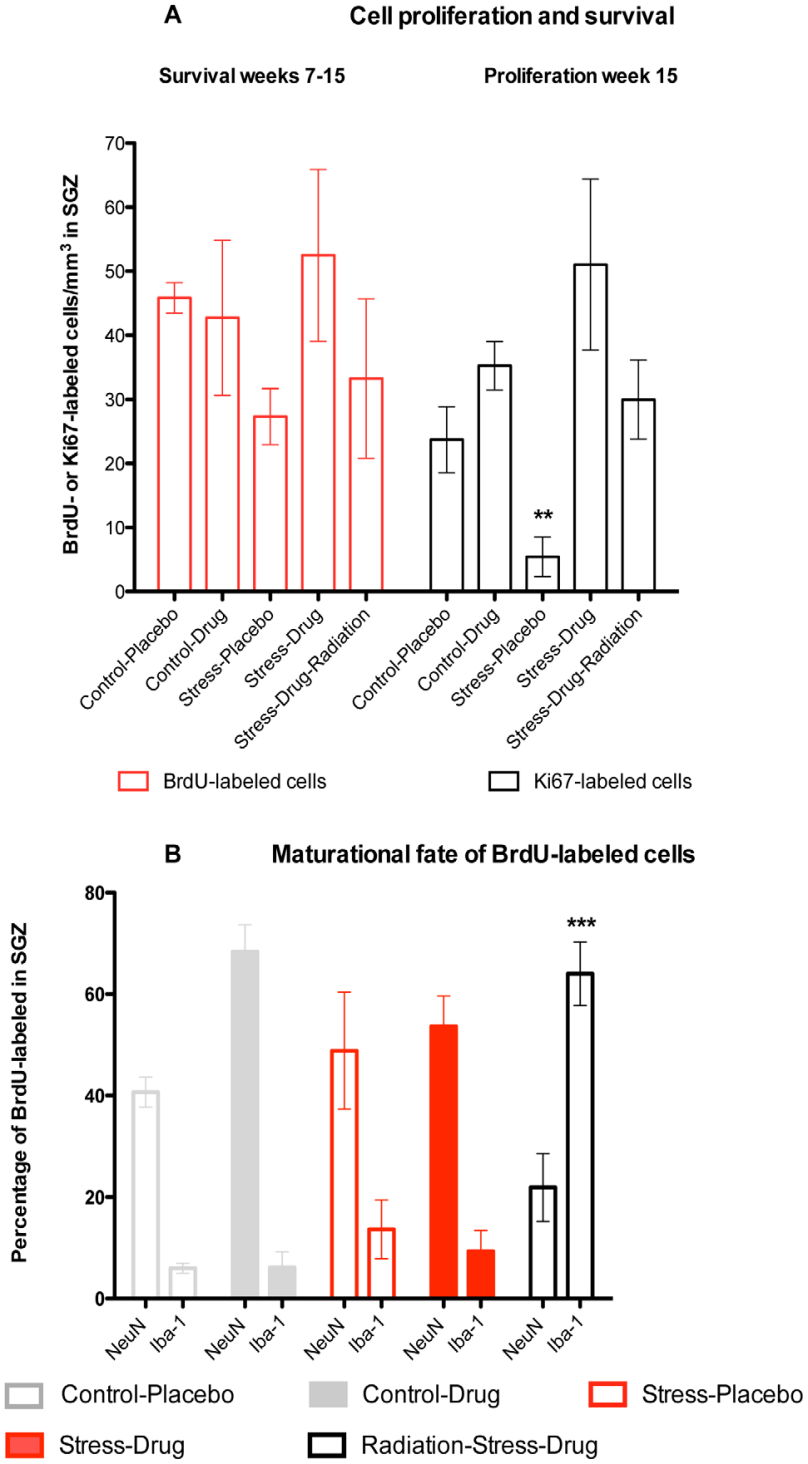
a. Left panel: Precursor cell survival rates. BrdU-labeling represented proliferating hippocampal precursors that took up BrdU on week-7 and survived until sacrifice on week-15. Log_10_ transformed rates of BrdU-labeled cells in the SGZ did not show an effect of experimental group (F_4,13_ = 1.7, p = 0.46). b. Right panel: Precursor cell proliferation rates. Hippocampal cell proliferation rates at the time of sacrifice were identified by the expression of Ki67. Log_10_ transformed rates of Ki67 expressing cells showed an overall effect of group (F_4,13_ = 6.8, p<0.001) stemming from decreased counts in Stress-Placebo compared to all other groups per Bonferroni post-hoc tests (p<0.01). b Left panel: Maturational fate of BrdU-labeled cells on week 7. The percentage of BrdU-labeled cells that co-labeled with NeuN was designated as new neurons and BrdU-labeled cells that co-labeled with Iba-1 were designated as microglia. Two-way ANOVA showed an overall interaction between group and maturational stage (P<0.0001), as well as an effect of group (p = 0.036), and maturational fate (p<0.0001). Bonferroni post-hoc tests showed greater levels of BrdU-NeuN co-labeling (p<0.01) and lower levels of BrdU-Iba-1 co-labeling (P<0.001) in the 4 non-irradiated subjects (Control-Drug, Control-Placebo, Stress-Drug, and Stress-Placebo) compared to irradiated subjects (Radiation-Stress-Drug group).

The rates of cell proliferation at the time of sacrifice (Ki67-expressing cells) was decreased in the Stress-Placebo group (p<0.01) when compared to Control-Placebo, while subjects treated with fluoxetine (Stress-Drug, Control-Drug) showed modest increases and the Radiation-Stress-Drug group showed normal rates (Fig. 4A). There were no significant group differences in precursor survival (BrdU-labeled cells) across all groups (Fig. 4A). The maturational speed ([number of DCX-expressing neurons at Stage 3 of maturation]/[total DCX-expressing neurons]) of Drug-treated groups (Control-Drug and Stress-Drug) was significantly increased when compared to Placebo groups (Control-Placebo and Stress-Placebo), and to the Irradiation-Stress-Drug group (p<0.001) (Fig. S2A). In terms of maturational fate of proliferating cells in the non-irradiated subjects (Control-Drug, Control-Placebo, Stress-Drug, Stress-Placebo), 52.9%±14.9% of BrdU-labeled cells co-labeled for the neuronal marker NeuN, compared to a significant decrease of 21.2%±11.6% in irradiated subjects (Radiation-Stress-Drug) (p<0.01) (Fig. 4B). By contrast, 64.0%±8.9% of BrdU-labeled cells in the irradiated subjects expressed Iba-1 and displayed microglial morphology compared to just 8.9%±7.5% in the non-irradiated subjects (Fig. 4B), which were shown to be significantly different (p<0.0001). There was no increase in astrocytosis, apoptosis, or necrosis per H & E staining and Hoechst staining (not shown) nor higher rates of microglial cells (Iba-1 positive, BrdU-negative) cells in the Radiation-Stress-Drug group compared to the Stress-Drug subjects (Fig. S2B).

### Correlation between behavior and histology

**Figure 5:**
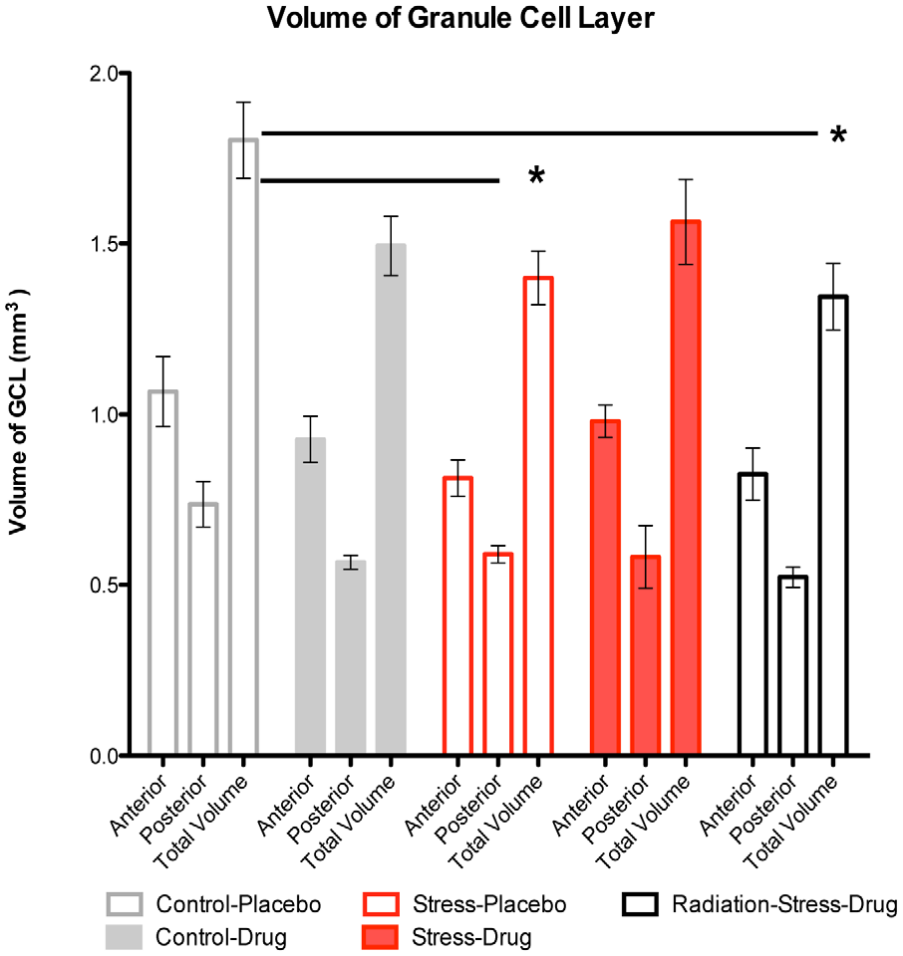
Volume of granule cell layer. Two-way ANOVA with Bonferroni post-hoc comparisons showed a significant decrease in granule cell layer volumes of Stress-Placebo and Radiation-Stress-Drug groups when compared with control-placebo (p<0.01).

We conducted linear regression analysis of correlations between anhedonia ratings and neurogenesis rates including all five groups (Fig. 3B). Anhedonia scores at each of the five different observational periods served as the dependent variable and the six subdivisions of neurogenesis rates served as independent variables. The analyses revealed that anhedonia scores at the final assessment block (weeks 13–15) inversely correlated with the density of new neurons that were in the anterior dentate gyrus and at Stage 3 of development (r^2^ = 0.67, p<0.0001) (Fig. 3B). By contrast, the number of immature Stage 1 and 2 neurons in the anterior dentate gyrus and the number of neurons at any maturational stage in the posterior dentate gyrus did not correlate with anhedonia scores at any of the behavioral assessment periods. Anhedonia scores at weeks 10–12 inversely correlated with the density of BrdU-NeuN co-labeled cells at Stage 4 of development in the anterior dentate gyrus (r^2^ = 0.28, p = 0.02) (Fig. 3B). The only other histological measure associated with behavioral scores was the rates of c-Fos expression in the anterior dentate gyrus, shown by the density of c-Fos-BrdU co-labeled cells (Fig. S3A), which positively correlated with anhedonia scores at the final time point during weeks 13–15 (r^2^ = 0.31, p = 0.008) (Fig. S3B). In terms of GCL volume, there were no overall group differences, but Bonferroni post hoc comparisons indicated significant volume reductions in the Stress-Placebo and Radiation-Stress-Drug (p<0.01) groups when compared with Control-Placebo monkeys (Fig. 5).

## Discussion

Our results show that bonnet macaques exposed to repeated social separation stress display depression-like features associated with suppression of hippocampal neurogenesis. Treatment with the antidepressant fluoxetine blocked emergence of depressive behaviors and stimulated neurogenesis. Ablating neurogenesis with temporal lobe irradiation abolished the salutary effects of the antidepressant and led to depression-like behavior in response to chronic stress. Together, these results provide the first evidence that hippocampal neurogenesis may play a role in the treatment of depression in NHPs similar to previous findings in rodents [19].

By using a nonhuman primate paradigm, we had the opportunity to more effectively replicate depression-related behaviors. The bonnet macaques exposed to repeated social separation stress displayed increases in anhedonia that involved a cluster of symptoms typically seen in depressive monkeys [33], including macaques [34]. Critically, this behavioral profile possesses significant face validity as an analog of clinical anhedonia, a core symptom of major depression [35]. The increases in the anhedonia scores were accompanied by decreases in hierarchy scores. Social subordinance is a hallmark of both chronic anxiety and depression in monkeys [33], [36]. This behavior of the NHPs in the setting of repeated social separation stress parallels clinical depression, as interpersonal loss is the predominant trigger of depression in humans [35] and chronic stress is a major epidemiological risk factor for major depression and chronic anxiety disorders [37].

The animals treated with fluoxetine did not demonstrate anhedonia-related behaviors. This is consistent with its therapeutic effect and with previous findings that sertraline, another SSRI, ameliorated anxiety-related behaviors and alcohol abuse in rhesus macaques that were exposed to repeated separation stress [17]. The therapeutic effects of fluoxetine in the stressed animals could not be explained by medication side effects because behavioral ratings in drug- and placebo-treated non-stressed controls did not differ. These behavioral changes did not result from emotional indifference, a potential side effect of fluoxetine treatment [38], because the drug-treated animals showed the same level of distress, in the form of affiliation, as placebo-treated subjects at the time of social reunion. Affiliation, an indicator of acute anxiety in bonnet macaques [13], increased not only during reunions but also during acute intruder stress. In summary, fluoxetine treatment specifically prevented stress-induced depressive behavior (anhedonia) and chronic anxiety behavior (subordinance), but had no impact on acute anxiety (affiliation). These behavioral effects of the medication are consistent with its therapeutic profile in humans [39].

Fluoxetine treatment had a profound effect on neurogenesis: non-irradiated fluoxetine-treated animals showed high rates of newly formed neurons at all stages of maturation in both the anterior and posterior dentate gyrus compared to their placebo-treated and irradiated counterparts. Fluoxetine treatment seems to have induced neurogenesis by increasing the speed of neuronal maturation, similar to its effects in mice [18], without increasing precursor cell survival (BrdU-labeled cells) or proliferation (Ki67-expressing cells). This is in contrast to the significant increase in proliferation and 600% increase in neurogenesis seen after ECT [16], and parallels the more robust clinical efficacy of ECT in treating depression compared to pharmacological agents such as fluoxetine [40]. Moreover, in the present study, chronic stress suppressed neurogenesis and cell proliferation in general, a pattern previously reported in adult rodents and tree shrews exposed to chronic stress [3], [4]. This is in contrast with recent evidence in rodents that chronic stress reduces the level of neuronal differentiation without decreasing proliferation rates [41].

Despite dramatic increases in new neurons following fluoxetine treatment, only a subpopulation of these, the Stage 3 DCX-expressing cells located in the anterior dentate gyrus, correlated with behavioral changes (anhedonia and hierarchy). The anatomic location of these new neurons in the anterior dentate gyrus is intriguing in light of the evidence that the anterior (ventral or temporal in rodents) hippocampus specifically mediates limbic behavior [50] via anatomic connections with the amygdala and prefrontal subregions [51] and supports the hypothesis that the antidepressant effect is linked with neurogenesis only in the anterior region of the dentate gyrus [52]. Likewise, neurogenesis rates in the posterior dentate gyrus failed to correlate with depressive behavior, consistent with the notion that the posterior hippocampus (dorsal or septal in rodents) mainly mediates spatial memory [53].

The specific association between depressive behavior and Stage 3 neurons was surprising, however. Although these cells had mature dendrites, they expressed immature neuronal markers (DCX and TUC-4), but not NeuN. They also failed to express c-Fos, suggesting that they did not participate in activation of synaptic circuitry, as well as the NR2B subunit of the NMDA receptor. These findings suggest that the Stage 3 granule cells had not entered the hyperplastic stage of neuronal maturation described in the adult mouse hippocampus. During this stage, at 4–6 weeks of age, differentiating granule cells enter a period of enhanced synaptic plasticity associated with NR2B expression and a lower threshold for LTP [26]. External stimuli preferentially activate these hyperplastic new neurons over less excitable mature granule cells [46]. Although presumed to be 6–7 weeks old, the Stage 3 neurons in the bonnet macaque seemed be less mature than the hyperplastic cells described in the mouse [26], and resembled younger, 3-week-old, mouse granule cells [47], reflecting the fact that duration of neuronal maturation in monkeys is 2–3 fold longer than in rodents [11]. Nonetheless, the fact that granule cells that were presumably 6–7 weeks old displayed an immature histological profile is surprising because in our previous study approximately 50% of 4-week old granule cells expressed NeuN following ECS treatment [16], and this rate of maturation is consistent with several other studies in nonhuman primates [14], [44], [48], [49]. It is possible that primate neuronal maturation takes many months because TUC-4-BrdU co-labeling has been detected ∼100 days post-BrdU injection [29]. On the other hand, it has been shown that TUC-4 can occasionally persist in mature neurons in the monkey brain [44].

The fact that behavioral changes correlated with the density of newly-formed Stage 3 neurons that had not yet entered the hyperplastic stage suggest that any role in mood regulation would have to involve indirect mechanisms. This role may involve reducing nonspecific activation in the dentate gyrus, since the rate of biochemical synaptic activation (c-Fos expression) of mature granule cells in the anterior dentate gyrus was reduced in bonnet macaques with high rates of neurogenesis and positively correlated with increases in depressive behavior (anhedonia ratings). This potential role is supported by a study where depressive behavior produced by bulbectomy in rats was associated with increased hippocampal c-Fos expression, while the reduction of depression with fluoxetine treatment decreased hippocampal c-Fos expression [54]. Neurogenesis may also play a role in reducing interference of older memories, thereby facilitating the acquisition of novel experiences, as it has been proposed to occur in rodents [55]. The reduction of synaptic excitability of mature granule cells by immature neurons provides a putative mechanism by which this reduction of interference may occur.

In the irradiation group, the animals showed increases in anhedonia and decreases in hierarchical behaviors despite antidepressant treatment, similar to the Stress-Placebo group. Although irradiation was administered at the beginning of the study, these animals did not display sustained increases in depressive behaviors until they were exposed to several weeks of stress. There was, however, a transient increase in anhedonia scores immediately following irradiation prior to the initiation of separation stress. These symptoms appear to have resulted from sedation or side effects of irradiation because the symptoms increased acutely (not gradually), were unaccompanied by hierarchical changes, and dissipated within 3-weeks, which is the typical duration of acute behavioral side effects of irradiation in mice [19].

The depressive behavior seen in the irradiated animals was associated with depressed levels of neurogenesis and altered maturational fate (the majority of the BrdU-labeled cells differentiated into microglia rather than neurons). The neurosuppressive effect of irradiation was subtler in the monkeys than in mice, where irradiation abolished all possibility of neurogenesis for months [18], [19]. In our subjects, cell proliferation resumed by weeks 13–16. Cell proliferation rates in the irradiated monkeys were normal by the time of sacrifice, with a majority of newly formed cells expressing Iba-1 and displaying microglial morphology. The total number of microglia, however, remained unchanged. Cell proliferation probably resumed over the 19 weeks between irradiation and sacrifice, likely potentiated by fluoxetine treatment in the interim. Irradiation may have reduced neuronal differentiation of mitotic stem cells, as shown by Steele and Lange [45], possibly by delaying the commitment of precursor cells to neuronal fate, as recently reported by our collaborators (Dranovsky, Hen et al., Science, *in press*). The predominance of microglia in this population raises the question of whether these mitotic cells represented an inflammatory reaction to irradiation, as has been reported in rodents [42]. Reduced neurogenesis accompanied by microglial proliferation has also been reported in mice [43] and rhesus macaques [44] exposed to brain injury. That these cells represent an inflammatory reaction is unlikely since both H & E staining and Hoechst staining failed to show evidence of increased astrocytosis, apoptosis, or necrosis in the irradiated hippocampi, and the total number of microglia was unaffected. This suggests that even if inflammatory macrophagic extravasation took place, and gave way to proliferation and differentiation into new microglial cells as late as weeks 7–8 (time of BrdU injection), then the stimulating inflammation response must have resolved by the time of sacrifice (week 15).

Because the method of ablation used in this study was relatively nonspecific, it was important to ascertain that the depressive behavior detected in the Radiated-Stress-Drug group did not result from delayed complications of irradiation such as inflammation and necrosis [42], [56]. To this end, we verified that the salient behavioral differences in the bonnet macaques occurred immediately prior to the time of sacrifice (week 13–15), at which point there was no evidence of inflammation or necrosis in the irradiated hippocampi despite extensive histopathological inspection. Therefore, we conclude that the loss of antidepressant efficacy in the irradiated monkeys most likely resulted from the reduction of neurogenesis.

In addition to reducing neurogenesis rates, chronic stress also reduced hippocampal granule cell layer (GCL) volume by 22% in the Stress-Placebo group and by 25% in the Radiation-Stress-Drug group, as compared with the Control-Placebo group. This is in agreement with previous research in rats [3], [32], and tree shrews [4]. Only one primate study has examined GCL volume after prenatal stress and found significant reductions in juvenile rhesus macaques [15]. Fluoxetine, on the other hand, may have prevented stress-induced reduction in GCL volume, as GCL dimensions in the Stress-Drug group did not differ from those of Control-Placebo subjects. This finding is in agreement with the prevention of stress-related shrinkage in GCL volume in tree shrews treated with the antidepressant tianeptine [4]. Temporal lobe irradiation abolished any protective effect of fluoxetine and decreased GCL volume in the Radiation-Stress-Drug group. This was not surprising because irradiation has been shown to decrease brain volume in fetal rhesus monkeys [57].

The interpretation of these data needs to be tempered by the small sample size. This is an unavoidable problem intrinsic to nonhuman primate research because of the limited availability of subjects. Nevertheless, conducting this experiment in monkeys revealed subtleties in the association between hippocampal neurogenesis and limbic behavior that not could not be detected in rodents [3], [19], for instance, that the regulation of neurogenesis was associated with changes only in depressive (anhedonia) and chronic-anxiety (subordinance) symptoms and was unrelated to changes in acute anxiety phenomena (affiliation and vigilance). Using NHPs also allowed us to localize and stage the population of new neurons correlated to behavioral changes in the primate brain, which differs in both anatomy and cellular kinetics to that of the rodent.

An outstanding question that warrants investigation is whether suppression of neurogenesis was sufficient to produce depressive behavior in the absence of stress. Although neurogenesis was acutely suppressed in the irradiated group, appearance of depressive behavior was delayed by several weeks. This suggests that neuro-suppression *per se* was not sufficient for producing depression in the irradiated monkeys and that exposure to several weeks of chronic stress was also needed. This view is supported by studies in which chronic uncontrollable stress consistently suppresses neurogenesis in rats [58], but only a fraction of these animals developed learned helplessness [59]. Although suppression of neurogenesis seemed insufficient to produce acute depression-like behavior in the irradiated animals, the later appearance of these behaviors despite fluoxetine treatment demonstrate that stimulating neurogenesis was necessary for mediating antidepressant efficacy. This has also been reported in mouse studies, in which ablating hippocampal neurogenesis did not produce behavioral abnormalities at baseline (in the absence of treatment), but abolished the therapeutic effects of antidepressant treatment in the setting of stressful conditions [18], [19].

Another outstanding issue, highlighted previously by Wang et al. [18] that future studies must address is whether neurogenesis plays a similar role in the treatment of preexisting depressive symptoms (similar to its clinical application), as it does in the prevention of depression during chronic stress. While the mechanisms needed to prevent depression are likely similar to those critical for reversing depression, it will be necessary to confirm this with a specifically-tailored study design. Future studies should also examine whether the timing of antidepressant response correlates with induction of neurogenesis. From our data, we assume that Stage 3 neurons take 6–7 weeks to develop in monkeys and maybe longer (perhaps 8–9 weeks) in humans. This implies that antidepressants would need at least 2-months to up-regulate this population in depressed patients. This speed of induction is too long to explain initial *response* to antidepressants, but is more consistent with the 8–10 weeks needed for complete *remission* of clinical depression [39]. There is some evidence that clinical remission is signaled by cognitive improvements that appear weeks after the initial response [60]. Interestingly, blocking hippocampal neurogenesis disrupts analogous cognitive function in rodents [61] several weeks after ablation [62]. Therefore, it is conceivable that hippocampal neurogenesis mediates delayed cognitive improvements associated with remission of depression but has no role in initial affective response to treatment (see review by Perera et al. [63]).

This study provides strong support for a link between the treatment of depression and the regulation of a specific population of new hippocampal neurons. The fact that this relationship was demonstrated in a plausible model of depression in anthropoid monkeys lends strong support for a similar role for neurogenesis in humans. Future studies are needed to confirm and expand these findings. If they do, new hippocampal neurons may serve as a marker of risk for depression and provide a tangible target for developing more effective and tolerable antidepressants.

## Supporting Information

Figure S1 a. Hierarchy scores in control subjects. In the absence of stress, the hierarchy scores and rank did not differ throughout the 15-week period of testing. b. Anhedonia scores in irradiation (XRT) pen. The XRT pen housed subjects that were irradiated (Radiation-Stress-Drug group, n = 4) and matched cage-mates that received sham irradiation (anesthesia only) (Stress-Drug group, n = 2). Compared to the control-placebo group, the irradiated subjects showed increases in anhedonia ratings at two time points: during the 3-week baseline period (immediately following irradiation, prior to stress/drug exposure) and during weeks 10–15 of stress/drug exposure (p<0.05). c. Affiliation scores during acute intruder stress on week 15. Intruder stress at week-15 increased affiliative behavior in the Stress-Placebo and Stress-Drug groups compared to Control-Placebo groups (p<0.05). There was no effect of drug treatment.
(TIF)

Figure S2 a. Maturational speed: Drug Vs. Placebo.The maturational speed was calculated as the percentage of DCX-expressing neurons with mature dendrites (Stage 3) among all DCX-expressing cells (Stages 1–3). One-way ANOVA showed an overall effect of group (p = 0.001) and Bonferroni's multiple comparison post-hoc test showed that the fraction of DCX Stage 3 cells was significantly higher in the drug-treated groups (Control-Drug and Stress-Drug) compared to Placebo-treated groups (Control-Placebo and Stress-Placebo), and to irradiated subjects (Radiation-Stress-Drug) (p<0.05). b. Total number of microglia. The total number of microglia did not differ between irradiated subjects and non-irradiated subjects (p = 0.8127). c. Fluorescent images of microglial cells. Images of newly-generated microglia where fluorescent images Iba-1-expressing cells (red), BrdU-expressing cells (green), and DAPI-expressing cells (blue) were overlayed (yellow).
(TIF)

Figure S3 a. c-Fos-expressing granule cells in the anterior dentate gyrus. The density of c-Fos-BrdU co-labeled neurons (mm3) in the anterior SGZ did not differ across all groups (p = 0.16).b. Correlation between Anhedonia scores and c-Fos expression in the anterior dentate gyrus. Anhedonia scores on weeks 13–15 correlated with increases in c-Fos expression in the anterior dentate gyrus (r^2^ = 0.31, p = 0.008), but not with c-Fos expression in the posterior dentate gyrus (not shown).
(TIF)
