# Supplementary figures and images for: Necessity of Hippocampal Neurogenesis for the Therapeutic Action of Antidepressants in Adult Nonhuman Primates
Source: PLoS One. 2011 Apr 15;6(4):e17600. doi: 10.1371/journal.pone.0017600 (PMC3078107; doi:10.1371/journal.pone.0017600)

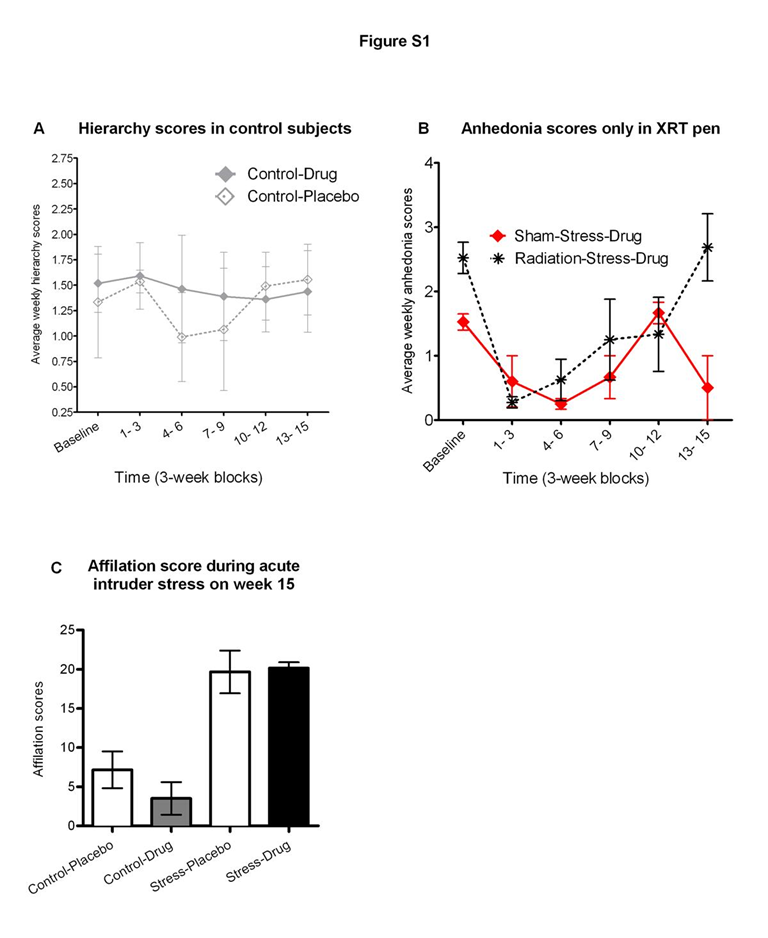

Supplement: Figure S1 — a. Hierarchy scores in control subjects. In the absence of stress, the hierarchy scores and rank did not differ throughout the 15-week period of testing. b. Anhedonia scores in irradiation (XRT) pen. The XRT pen housed subjects that were irradiated (Radiation-Stress-Drug group, n = 4) and matched cage-mates that received sham irradiation (anesthesia only) (Stress-Drug group, n = 2). Compared to the control-placebo group, the irradiated subjects showed increases in anhedonia ratings at two time points: during the 3-week baseline period (immediately following irradiation, prior to stress/drug exposure) and during weeks 10–15 of stress/drug exposure (p<0.05). c. Affiliation scores during acute intruder stress on week 15. Intruder stress at week-15 increased affiliative behavior in the Stress-Placebo and Stress-Drug groups compared to Control-Placebo groups (p<0.05). There was no effect of drug treatment. (TIF) [file pone.0017600.s001.tif]

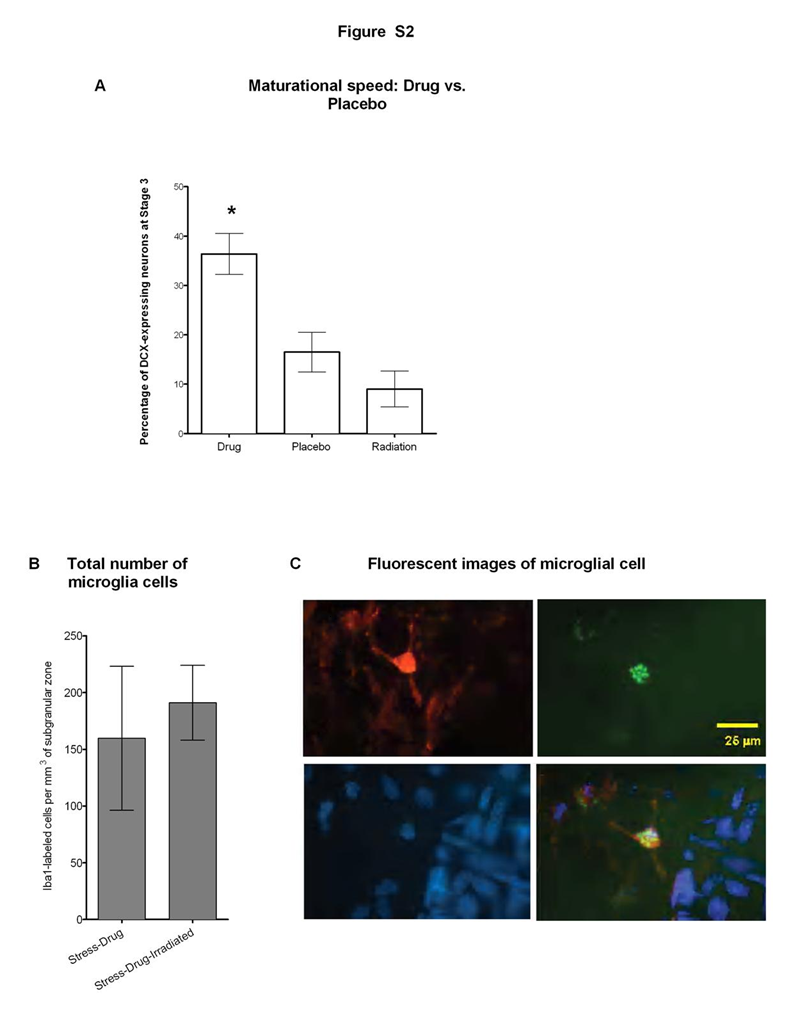

Supplement: Figure S2 — a. Maturational speed: Drug Vs. Placebo.The maturational speed was calculated as the percentage of DCX-expressing neurons with mature dendrites (Stage 3) among all DCX-expressing cells (Stages 1–3). One-way ANOVA showed an overall effect of group (p = 0.001) and Bonferroni's multiple comparison post-hoc test showed that the fraction of DCX Stage 3 cells was significantly higher in the drug-treated groups (Control-Drug and Stress-Drug) compared to Placebo-treated groups (Control-Placebo and Stress-Placebo), and to irradiated subjects (Radiation-Stress-Drug) (p<0.05). b. Total number of microglia. The total number of microglia did not differ between irradiated subjects and non-irradiated subjects (p = 0.8127). c. Fluorescent images of microglial cells. Images of newly-generated microglia where fluorescent images Iba-1-expressing cells (red), BrdU-expressing cells (green), and DAPI-expressing cells (blue) were overlayed (yellow). (TIF) [file pone.0017600.s002.tif]

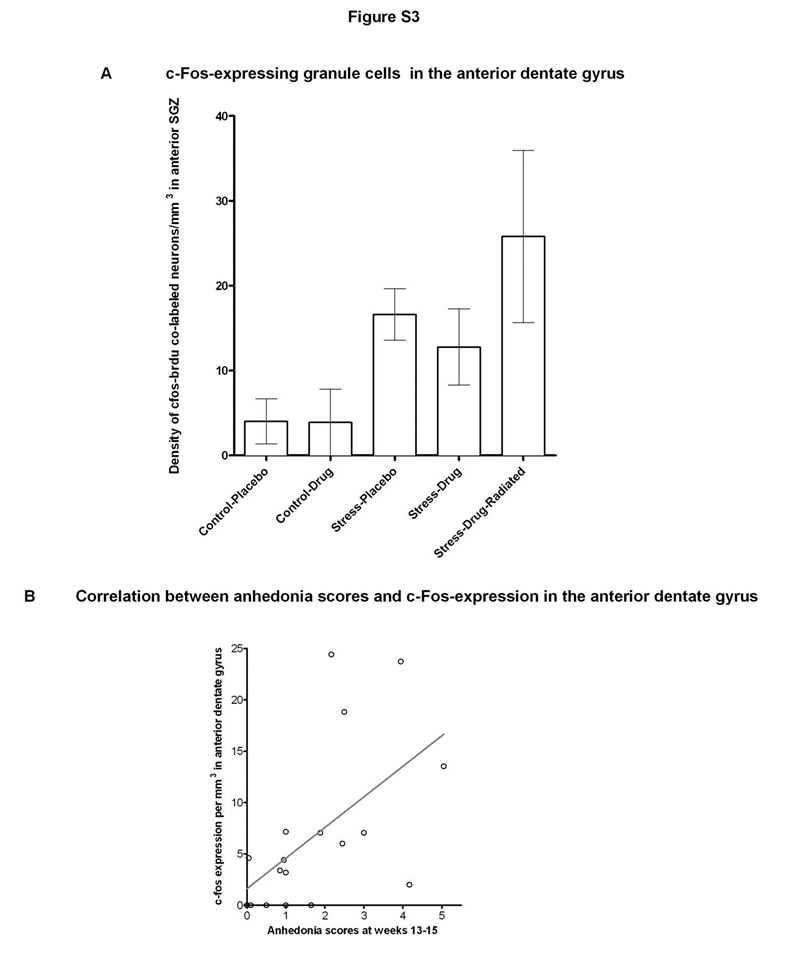

Supplement: Figure S3 — a. c-Fos-expressing granule cells in the anterior dentate gyrus. The density of c-Fos-BrdU co-labeled neurons (mm3) in the anterior SGZ did not differ across all groups (p = 0.16).b. Correlation between Anhedonia scores and c-Fos expression in the anterior dentate gyrus. Anhedonia scores on weeks 13–15 correlated with increases in c-Fos expression in the anterior dentate gyrus (r2 = 0.31, p = 0.008), but not with c-Fos expression in the posterior dentate gyrus (not shown). (TIF) [file pone.0017600.s003.tif]
